# Investigation on the Correlation between Dispersion Characteristics at Terahertz Range and Dielectric Permittivity at Low Frequency of Epoxy Resin Nanocomposites

**DOI:** 10.3390/polym14040827

**Published:** 2022-02-21

**Authors:** Ze Lian, Danyang Chen, Shengtao Li

**Affiliations:** 1 State Grid Shanxi Electric Power Research Institute, Taiyuan 030001, China; cdydanyangchen@hotmail.com; 2 State Key Laboratory of Electrical Insulation and Power Equipment, Xi’an Jiaotong University, Xi’an 710049, China; sli@mail.xjtu.edu.cn

**Keywords:** epoxy resin, nanocomposites, terahertz time-domain spectroscopy, Lorentz oscillator model, dispersion characteristics

## Abstract

Despite the extensive research on nanocomposites, a fundamental theory on the interface region is still difficult to achieve. In the present paper, we chose epoxy resin and nano-SiO_2_, nano-SiC, nano-ZnO to prepare three kinds of nanocomposites. The dispersion characteristics at the terahertz range and dielectric permittivity at 1 Hz of epoxy resin-based nanocomposites were investigated. The reduction of the permittivity of nanocomposites at a slight filler concentration was absent at the terahertz range. The measurement results at 1 Hz show that the interaction between nano-SiO_2_, nano-SiC particles and epoxy resin was strong with the modification of the silane coupling agent. However, the modification of nano-ZnO particles was invalid. The Lorentz harmonic oscillator model was employed to fit the dispersion characteristics. The relevance between the damping constant and the dielectric permittivity at low frequency was established, indicating that the increase in the damping coefficient results from the restriction of the molecular chain motion by the interfacial region. The present results in this paper reveal a bright prospect of terahertz time-domain spectroscopy in establishing the theory of nanocomposite dielectric.

## 1. Introduction

Since the concept of “nanodielectrics” was first introduced in 1994 by Lewis, it has attracted much attention due to its superior properties and excellent prospect in the electric industry [1,2,3]. Various enhanced properties of nanocomposites, such as electric strength, mechanical strength, space charge accumulation, and so on, were reported by researchers [4,5,6,7]. Nowadays, it has been generally accepted that most of the superior properties can be attributed to the interface region [8,9]. Although several models were proposed to interpret the superior properties [2,10,11,12], it is still difficult to accomplish a fundamental understanding of the interaction between nanoparticles and poly matrices [8,13]. 

With the addition of a nano-filler, the molecular motion of polymer is suppressed by the interface region, resulting in the relaxation processes at different frequency ranges exhibiting different changes with the electric field [14]. However, the relationship between the dielectric properties and the microstructure has not been established [9]. According to the proposed models, the thickness of the interface region is estimated to be about tens to hundreds of nanometers, depending on filler type, filler size, and the interaction between the nano-filler and the polymer matrix [11]. Due to the characteristic frequency of this space scale being located in the high-frequency region, the dispersion characteristics at the high-frequency region may provide a glimpse of the change in microstructure. The terahertz spectrum includes phonon vibration, small molecule rotation, hydrogen bond stretching torsion, and chemical bond low-frequency vibration, which cannot be characterized by a broadband dielectric spectrum at low frequency [15]. Significant progress has been achieved in investigating the molecular dynamics of polymers in the terahertz band [16,17,18,19,20]. The coherent detection mode of the terahertz time-domain spectroscopy (THz-TDS) makes it convenient to obtain the dispersion characteristics of testing materials at the terahertz range [21,22,23,24,25]. The investigation of the dispersion characteristics of nanocomposites is beneficial to the exploiting of fundamental theory in nanocomposites [26,27,28,29,30].

Among the polymer matrices that constitute nanocomposites, epoxy resin is one of the most popular materials for insulating systems [31,32]. It is widely used in dry-type transformers, generator stators, bushing, cable termination, and so on [33,34]. The epoxy resin-based nanocomposites, which were expected to be the next generation of dielectric materials, have attracted much attention in recent years [35]. In this paper, we employed epoxy resin as a polymer matrix and three kinds of nano-fillers to constitute nanocomposites. The THz-TDS was employed to study the dispersion characteristics of epoxy resin-based nanocomposites. The dielectric constant at low frequency (1 Hz in this paper) was obtained too. The correlation of dispersion characteristics at the terahertz range and dielectric constant at low frequency was discussed. 

## 2. Materials and Methods

### 2.1. Fabrication of Epoxy Resin Nanocomposites

In the present study, we chose bisphenol-A epoxy resin (E51) and methyl tetrahydrophthalic anhydride hardener as matrix and curing agent, respectively. The curing agent was used at 80 phr. DMP30 was chosen as the accelerant which was used at 1 phr. Three kinds of epoxy/nanocomposites were prepared with the addition of nano-SiO_2_ particles, nano-SiC particles and nano-ZnO particles with average diameter of 40 nm. These nanomaterials in this paper are commercial nanomaterials (Hangzhou Wanjing New Material Co., LTD, Hangzhou, China). The silane coupling agent (KH550) was used to ensure the dispersity of nanoparticles in epoxy resin matrix. 

**Figure 1:**
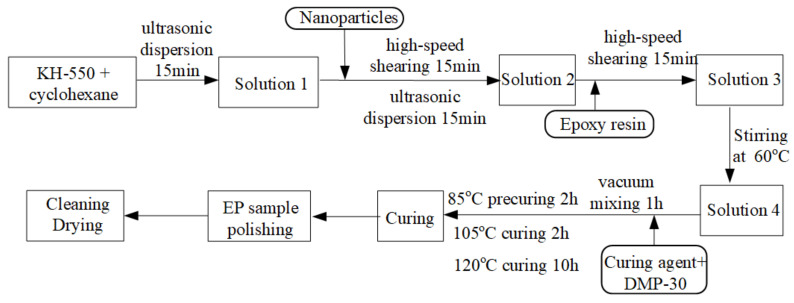
Sample preparation process of nanocomposites.

The fabrication process of epoxy resin nanocomposites was shown in Figure 1. During the fabrication process, we employed a shearing instrument IKA-T25 with a maximum speed of r/min (Shanghai Yikong Electromechanical Co., LTD, Shanghai, China) and an ultrasonic device KQ-100KDE (Kunshan Ultrasonic Instrument Co., LTD, Kunshan, China), which operates at a frequency of 40 kHz and a power of 99 W. The KH-550 and cyclohexane were mixed by ultrasonic dispersion. Then the nanoparticles were added to the mixture (solution 1). Both high-speed shearing and ultrasonic dispersion were applied on solution 1 to avoid the agglomeration of nanoparticles. After the mixture of epoxy resin, high-speed shearing was employed again. Then, the curing agent and the accelerant DMP30 were added to the mixture. The obtained mixture was stirring at in vacuum for 1 h at 60 °C. Then, to accomplish the curing process, the mixture was poured into the mold and placed into the oven at 85 °C for 2 h, 105 °C for 2 h, and 120 °C for 10 h, respectively. When the samples were cooled, the polishing, cleaning, and drying were carried out before the measurements. The arrangement of nanocomposites samples was listed in Table 1. It is generally accepted that the amount of nano-filler is lower than 10%. Therefore, we chose five kinds of filler content to constitute nanocomposites in the present paper, as listed in Table 1. The thickness of each sample was kept at about 1 mm.

### 2.2. Terahertz Time-Domain Spectroscopy System

**Figure 2:**
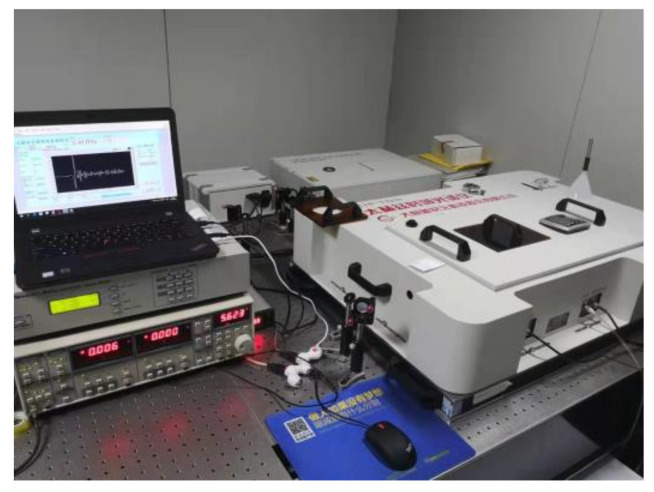
The CIP-TDS system employed in present paper.

A CIP-TDS terahertz time-domain spectroscopy system (DaHeng Technology Co., LTD, Beijing, China) of was employed to investigate the dispersion characteristics of nanocomposites at terahertz range. The terahertz time-domain spectroscopy system was shown in Figure 2. In the present paper, the experiments were carried out in transmission mode. As shown in Figure 2, it is mainly composed of femtosecond laser, terahertz radiation generation device, terahertz detection system, time delay control system and various lenses. In the present CIP-TDS systems, the femtosecond laser was emitted by a model-locked Ti:sapphire laser. The pulse width, wavelength and repetition rate of the laser were 80 fs, 800 nm and 80 MHz, respectively. The laser was split into two beams: one for pump of terahertz emission and the other for the detection of terahertz radiation [36]. In this study, the low temperature-grown GaAs photoconductive antennas and ZnTe crystal were employed for the emitter and detector of terahertz radiation, respectively. The system achieves a peak dynamic range of 60 dB and a bandwidth of 3.5 THz. In order to avoid the strong absorption effect of water vapor on terahertz waves, dry nitrogen was continuously introduced into the cavity to keep the relative humidity below 3% [37]. After obtaining the time-domain spectrum of the sample, the frequency domain spectrum was obtained by fast Fourier transform, and the dielectric constant and dielectric loss were calculated by using the refractive index and extinction coefficient in the transmission function [22]. Each sample was tested three times and the average value was taken as the final result.

In addition, in order to have a comparison with the dielectric properties at low frequency, we employed a Novocontrol broadband dielectric spectrometer to measure the dielectric constant at 1 Hz.

### 2.3. Methods

In the THz-TDS measurements, the reference signals and the sample signals in time-domain were measured, respectively. Then the fast Fourier transform was performed to obtain the *E_ref_* (*υ*) and *E_s_*(*υ*) in frequency domain [37]. The transmission function of sample for terahertz wave can be expressed as follows:

(1) $$H(\upsilon )=\frac{E_{s}(\upsilon )}{E_{ref}(\upsilon )}=\rho (\upsilon )e^{-i\Delta \phi (\upsilon )}$$

As described in the literature, we can acquire the refractive index and extinction coefficient of sample by the following equations [38,39]:

(2) $$n_{s}\left(\upsilon \right)=1+\frac{c\varphi \left(\upsilon \right)}{\upsilon d}$$

(3) $$\kappa_{s}\left(\upsilon \right)=\left\{\ln\left[\frac{4n_{s}\left(\upsilon \right)}{{\left(n_{s}\left(\upsilon \right)+1\right)}^{2}}\right]-\ln\rho \left(\upsilon \right)\right\}\frac{c}{\upsilon d}$$

where *c* is the speed of light in vacuum and *d* is the thickness of the sample. According to the generalized Maxwell relation between the complex permittivity and the complex refractive index, the real and imaginary parts of the permittivity are obtained:

(4) $$\varepsilon \prime \left(\upsilon \right)={\left[n_{s}\left(\upsilon \right)\right]}^{2}-{\left[\kappa_{s}\left(\upsilon \right)\right]}^{2}$$

(5) $$\varepsilon \prime \prime \left(\upsilon \right)=2n_{s}\left(\upsilon \right)\kappa_{s}\left(\upsilon \right)$$

The dispersion characteristics of epoxy resin nanocomposites in the terahertz range can be well described by the Lorentz oscillator model [40,41]:

(6) $$\varepsilon^{*}\left(\upsilon \right)=\varepsilon_{\infty }+\frac{\Omega^{2}}{\left(\upsilon_{0}^{2}-\upsilon^{2}\right)-i\gamma \upsilon }$$

where *ε*_∞_ is the dielectric constant in the infinite frequency, Ω, *υ*_0_ and *γ* are respectively the oscillator strength, the resonance frequency and the damping constant of the resonant mode.

## 3. Results

**Figure 3:**
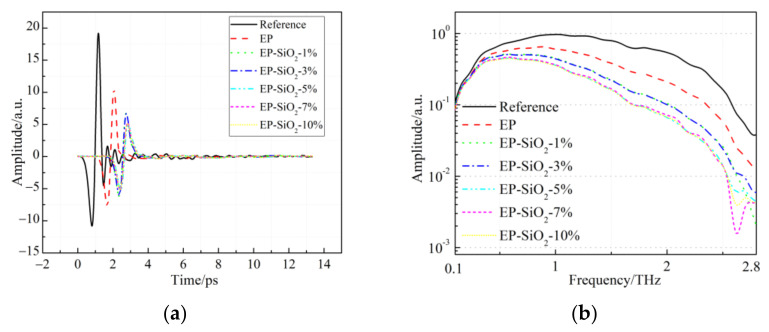
THz time-domain spectra and amplitude spectra of the reference and the EP/SiO_2_ nanocomposites: (**a**) Terahertz waves of reference and EP/SiO_2_ samples in time domain; (**b**) The corresponding spectra in frequency domain.

Figure 3a shows the measured signals of EP/SiO_2_ nanocomposites in the time domain. Compared with the reference signal, the signals changed markedly because of the interaction between the terahertz radiation and the samples. Moreover, the waveforms nanocomposites reach a maximum later than that of the epoxy resin. The corresponding amplitude spectra in the frequency range are given in Figure 3b. With the filler content increasing, the amplitude of the nanocomposites decreased slightly.

**Figure 4:**
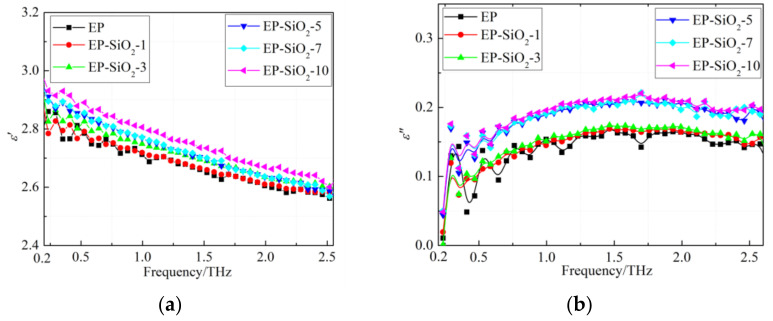
Frequency dependence of real and imaginary part of dielectric function of EP/SiO_2_ nanocomposites: (**a**) Real part; (**b**) Imaginary part.

According to the above formulas in Section 2.3, we can acquire the dielectric permittivity of the EP/SiO_2_ nanocomposites. Figure 4 exhibits the frequency dependence of the real and imaginary part dielectric function of the EP/SiO_2_ nanocomposites. As shown in Figure 4a, the dielectric permittivity of all samples decreases monotonically with an increasing frequency. In addition, at a certain frequency, the *ε*′ of EP/SiO_2_ nanocomposites gets higher when increasing the filler content. The imaginary part of the dielectric function is illustrated in Figure 4b. The results indicate that the *ε*″ of EP/SiO_2_ nanocomposites has no evident changes when filler content is lower than 3%. When the filler content is higher than 5%, the *ε*″ of nanocomposites becomes larger than the EP.

**Figure 5:**
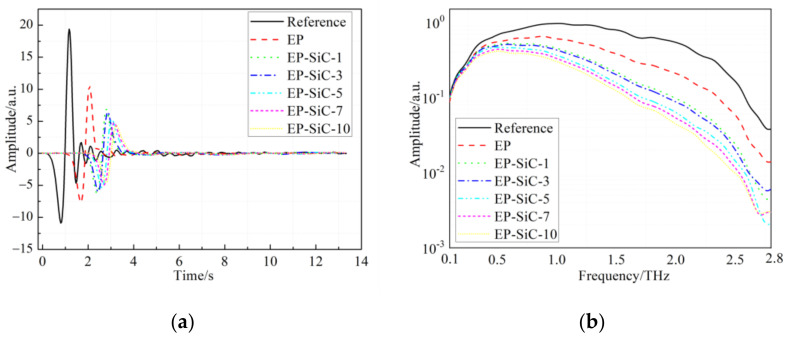
THz time-domain spectra and amplitude spectra of the reference and the EP/SiC nanocomposites: (**a**) Terahertz waves of reference and EP/SiC samples in time domain; (**b**) The corresponding spectra in frequency domain.

The same measurements were carried out on the EP/SiC nanocomposite. Figure 5 exhibits the waveforms of the reference and the EP/SiC nanocomposites in the time domain and the corresponding spectra in the frequency domain. Similarly, a distinct change can be observed in Figure 5a. The time delay of the maximum in the signals of the EP/SiC nanocomposites is bigger than that of the EP/SiO_2_ nanocomposites, which may result from a higher dielectric constant. Accordingly, the amplitude spectra show a monotonically decreasing relationship with respect to the filler content.

**Figure 6:**
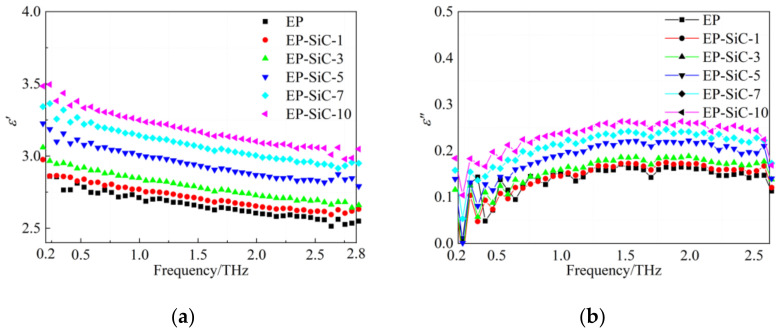
Frequency dependence of real and imaginary part of dielectric function of EP/SiC nanocomposites: (**a**) Real part; (**b**) Imaginary part.

Figure 6 illustrates the calculated dielectric permittivity of the EP/SiC nanocomposites. Figure 6a shows the frequency dependence of the real part of dielectric function in the 0.2–2.8 THz range. No peak was observed on the curves of the dielectric function. It is apparent that the *ε*′ of all the EP/SiC samples decreases monotonously with the increase in frequency. However, with the filler content increasing, the increase in the EP/SiC nanocomposites is more significant than that of the EP/SiO_2_ nanocomposites. As shown in Figure 6b, the *ε*″ of the tEP/SiC nanocomposites increases with the increasing filler content, which is different from the situation in the EP/SiO_2_ nanocomposites.

**Figure 7:**
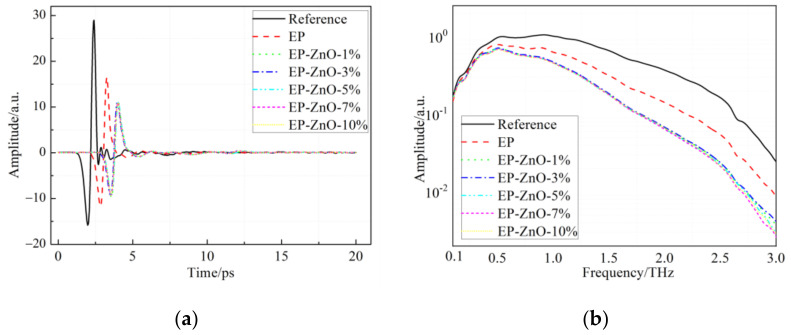
THz time-domain spectra and amplitude spectra of the reference and the EP/ZnO nanocomposites: (**a**) Terahertz waves of reference and EP/ZnO samples in time domain; (**b**) The corresponding spectra in frequency domain.

Figure 7 shows the THz time-domain waveforms and amplitude spectra of the reference and the EP/ZnO nanocomposites. The curves in Figure 7a exhibit similar characteristics compared to the results of the EP/SiO_2_ nanocomposites. Likewise, there is no peak appearing in the corresponding amplitude spectra, as demonstrated in Figure 7b.

**Figure 8:**
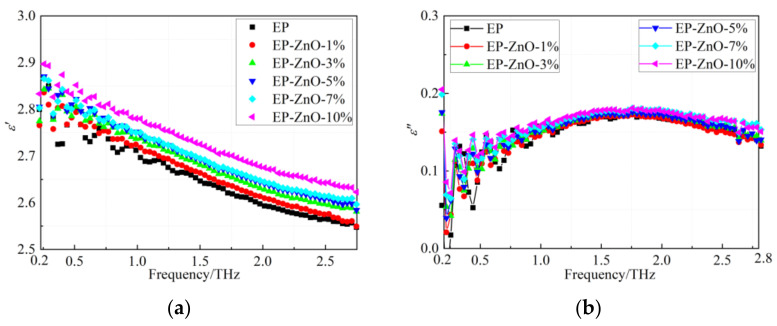
Frequency dependence of real and imaginary part of dielectric function of EP/ZnO nanocomposites: (**a**) Real part; (**b**) Imaginary part.

Similar to the two types of nanocomposites above, we can obtain the frequency dependence of the real and imaginary part of the dielectric function of the EP/ZnO nanocomposites, as shown in Figure 8. As demonstrated in Figure 8a, the addition of the nano-ZnO fillers makes a similar difference to the dielectric permittivity of the EP compared to the nano-SiO_2_ fillers. Nevertheless, the imaginary part of the dielectric permittivity of the EP/ZnO nanocomposites, as shown in Figure 8b, differs from that of both the EP/SiO_2_ and the EP/SiC nanocomposites. The curves shown in Figure 8b overlap each other. It is difficult to tell them apart. These results imply that the three types of nano-fillers employed in this study have different effects on the dispersion characteristics of the epoxy resin.

## 4. Discussion

**Figure 9:**
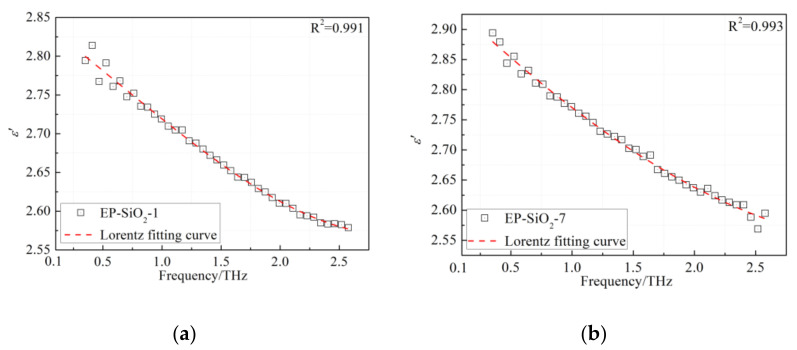
Fits based on Lorentz resonant mode: (**a**) Dielectric permittivity of the EP-SiO_2_-1 sample and the fitting curve with a confidence coefficient of 0.991; (**b**) Dielectric permittivity of the EP-SiO_2_-7 sample and the fitting curve with a confidence coefficient of 0.993.

In this paper, we fitted the dielectric function of the EP nanocomposites by the Lorentz oscillator model with one vibrational mode, as given in Equation (6). For simplicity, we only exhibit the fitting curves of the EP-SiO_2_-1 and the EP-SiO_2_-7 in this paper. As shown in Figure 9, the dielectric function of the EP/SiO_2_ nanocomposites can be well reproduced by the Lorentz oscillator model. For test results of both the EP-SiO_2_-1 and the EP-SiO_2_-7 samples, a confidence coefficient higher than 0.99 is acquired. The excellent agreement between the experimental data and fitting curves indicates that the dispersion characteristics of nanocomposites based on the epoxy resin in the 0.1–2.5 THz are dominated by the resonant process. The dielectric functions of the EP/SiO_2_, EP/SiC, and EP/ZnO nanocomposites were fitted with the same process. The obtained fitting parameters are summarized in Table 2. For all fitting processes, the confidence coefficient is larger than 0.98.

**Figure 10:**
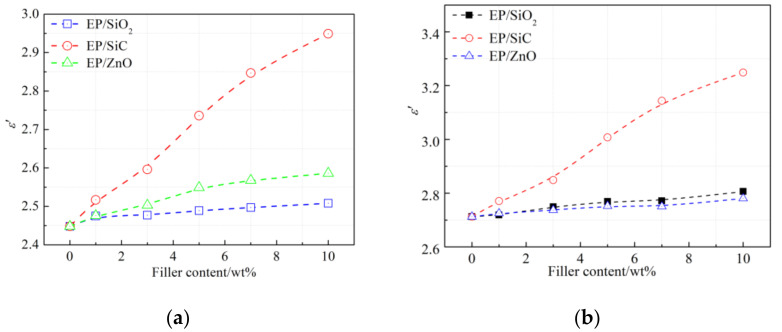
The dielectric permittivity for three types of nanocomposites with respect to filler content: (**a**) At infinite frequency; (**b**) At 1 THz.

In order to have a more intuitive understanding of the effect of nanoparticles on dispersion characteristics, we draw the dielectric permittivity of nanocomposites with respect to filler content at an infinite frequency (*ε*_∞_ in Table 2) and at 1 THz in Figure 10. As shown in Figure 10a, the dielectric permittivity at an infinite frequency increases with the introduction of nanofillers. At an infinite frequency, the effective medium theory is suitable to estimate the dielectric response of the composites. Therefore, the increase *ε*_∞_ of nanocomposites can be attributed to the fact that the nanofillers have higher dielectric permittivity than the epoxy resin matrix. The dielectric constant at a low frequency of SiO_2_, ZnO and SiC employed in this paper is 3.9, 4.5, and 9.8, respectively. Consequently, at the same filler content, the EP/SiC sample owns the highest dielectric permittivity and the EP/SiO_2_ owns the lowest. 

Figure 10b shows the dielectric permittivity of nanocomposites at 1 THz. Likewise, the *ε′* increases with the increasing filler content. The experimental phenomenon that dielectric permittivity at low frequencies decreases with the introduction of nanofillers were not observed. We reported similar results in a previous study [26]. In addition, the vibration and rotation of molecular groups are unable to keep up with the electromagnetic field, resulting in the dielectric permittivity at infinite frequency being slightly lower than that at 1 THz.

**Figure 11:**
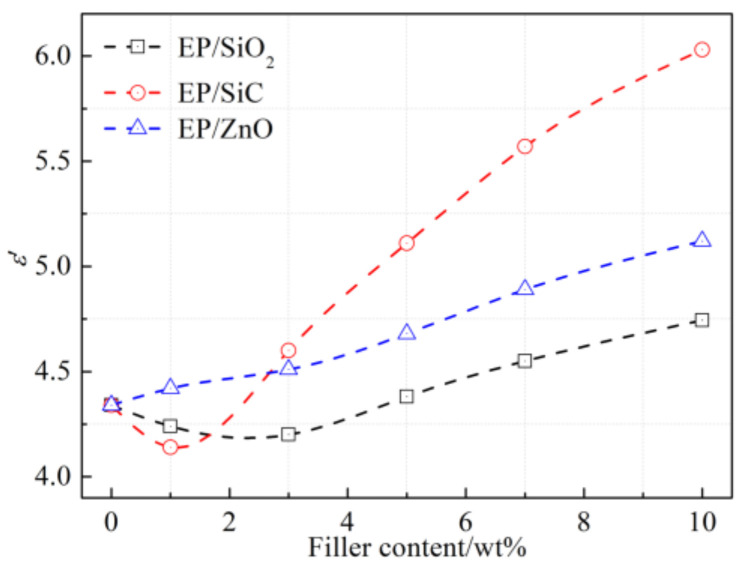
The dielectric permittivity at 1 Hz for three types of nanocomposites with respect to filler content.

The absence of the feature that dielectric permittivity of nanocomposites decreases after introducing nanofillers leads us to wonder whether there is a nano-structure formed between the nanofillers and the epoxy resin matrix in the composites studied in the present paper. We measured the dielectric permittivity at 1 Hz for the three types of nanocomposites, as shown in Figure 11. The results indicate that the dielectric permittivity of EP/SiO_2_ and EP/SiC decreases firstly and then it increases with the increasing filler content. The *ε′* of the EP/SiC achieves the minimum value at a filler content of 1%, while *ε′* of the EP/SiO_2_ achieves the minimum at 3%. However, the dielectric permittivity at 1 Hz of the EP/ZnO in the present study increases monotonously with the increasing filler content. In general, the reduction of dielectric permittivity was attributed to the suppression of molecular motion by the addition of nanofillers. 

According to our previous work [26], the dielectric permittivity of the epoxy resin at the 10^−1^ to 10^3^ Hz frequency range is dominated by the α–process, which is affected significantly by the interface region. The stronger the interaction between the nanofillers and epoxy resin is, the more effective the suppression of molecular motion works. Therefore, we can speculate that the interaction strength of the interface region in each nanocomposite is different. The surface of the nano-SiO_2_ and the nano-SiC were modified successfully, leading to the strong interaction between the polymer matrix and these nanoparticles. However, the modification of the silane coupling agent used in this study is invalid for nano-ZnO particles, resulting in the fact that the alignment layer did not appear in EP/ZnO nanocomposites.

**Figure 12:**
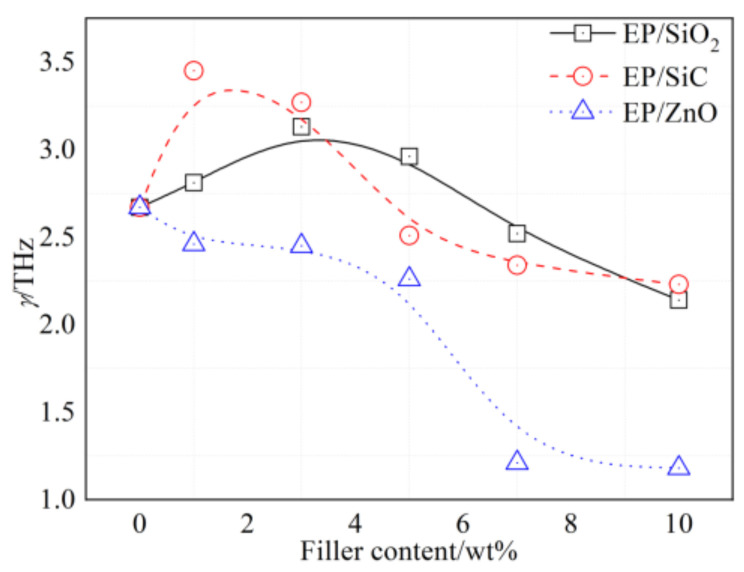
The damping constant for three types of nanocomposites versus filler content.

Due to that the *γ* representing the damping constant of the resonant mode, which can characterize the strength of molecular motion, we draw the fitted damping constant (as listed in Table 2) of the nanocomposites with respect to the filler content in Figure 12. As shown in Figure 12, the damping coefficients of EP/SiO_2_ and EP/SiC increase firstly and then decrease with the increase in filler content, which exhibits a converse trend with the dielectric constant at 1 Hz. When the filler content is greater than 7%, the *γ* of the nanocomposite is lower than that of the unfilled epoxy resin. This result indicates that the agglomeration of nanoparticles occurred in the nanocomposites with high filler content. Moreover, the *γ* of EP/SiO_2_ and EP/SiC reach their maximum at the filler content of 3% and 1%, respectively, which is identical with the filler content that they achieve their minimum permittivity.

As for EP/ZnO, the damping coefficient decreases monotonously with increasing filler content. This result implies that the introduction of nano-ZnO in the present study loosens the molecular motion of the epoxy resin matrix. Consequently, no decline appeared in the *ε′* of the EP/ZnO composites, as shown in Figure 11. The progressive increase with increasing filler content is mainly caused by the introduction of the ZnO particle, which owns a higher dielectric permittivity than the epoxy resin matrix and the loose structure resulting from the agglomeration of nanoparticles. 

Moreover, the results in Figure 12 indicate that the silane coupling agent KH550 improves the good adhesion between nano-SiO_2_ particles, nano-SiC particles, and the epoxy matrix, resulting in the motions of polymer main chains being restrained significantly. However, the improvement is absent in the EP/ZnO nanocomposites. Due to the strong relevance between the dielectric permittivity at 1 Hz and the damping constant, we can deduce that the damping constant of dispersion characteristics at the terahertz range can effectively describe the strength of molecular motion which contributes greatly to the dielectric permittivity at low frequencies. The bigger the damping constant is, the stronger the interaction between the nanofillers and the polymer matrix is. Hence, the study of dispersion characteristics at the terahertz range may provide valuable information for the fundamental understanding of interface regions in nanocomposites.

## 5. Conclusions

In this paper, we prepared three kinds of nanocomposites with different filler contents based on epoxy resin. The dispersion characteristics at the terahertz range of the nanocomposites were investigated with THz-TDS. We can draw the following conclusions:

(1) The addition of nano-SiO_2_ and nano-SiC have an obvious influence on the dispersion characteristics of the epoxy resin, while the addition of nano-ZnO does not make any difference. The employment of the silane coupling agent KH550 did not establish a strong interaction between the nano-ZnO particles and the epoxy resin matrix.
(2) The permittivity of the nanocomposites at 1 THz increases monotonously with the increase in nanoparticle content. Whereas the permittivity of the nanocomposites at 1 Hz shows different variation rules. The molecular motion of epoxy resin was suppressed by the addition of nano-SiO_2_ and nano-SiC with slight filler content.
(3) The dielectric function of nanocomposites at the terahertz range can be well reproduced by the Lorentz oscillator model. The fitted damping coefficient of nanocomposites with respect to filler content shows a converse trend to which the permittivity at 1 Hz varies with filler content.
(4) A strong relevance between the dielectric permittivity at low frequency and the damping constant was discovered in the present paper. These results show that THz-TDS is very promising in the study of the interface region and the fundamental understanding of nanocomposites.

## Figures and Tables

**Table 1:** Arrangement of nanocomposite samples.

Sample	Component	Filler content
EP/SiO_2_	Epoxy resin + 40 nm SiO_2_	0%,1%,3%,5%,7%,10%
EP/SiC	Epoxy resin + 40 nm SiC	0%,1%,3%,5%,7%,10%
EP/ZnO	Epoxy resin + 40 nm ZnO	0%,1%,3%,5%,7%,10%

**Table 2:** Lorentz fitting parameters for the dielectric functions of nanocomposites.

Composites	*ε*_∞_	*ω*_0_/2π (THz)	Ω/2π (THz)	*γ* (THz)
EP	2.448	1.732	1.41	2.67
EP/SiO_2_-1	2.475	1.828	1.85	2.81
EP/SiO_2_-3	2.477	1.774	1.79	3.13
EP/SiO_2_-5	2.489	1.721	1.42	2.96
EP/SiO_2_-7	2.497	1.692	1.26	2.52
EP/SiO_2_-10	2.508	1.671	1.09	2.14
EP/SiC-1	2.517	1.829	2.17	3.45
EP/SiC-3	2.596	1.796	2.09	3.27
EP/SiC-5	2.736	1.744	1.55	2.51
EP/SiC-7	2.847	1.742	1.34	2.34
EP/SiC-10	2.949	1.689	1.21	2.23
EP/ZnO-1	2.462	1.772	1.24	2.46
EP/ZnO-3	2.503	1.771	1.16	2.45
EP/ZnO-5	2.549	1.783	1.04	2.26
EP/ZnO-7	2.568	1.787	0.63	1.21
EP/ZnO-10	2.586	1.792	0.35	1.18

## Data Availability

The data presented in this study are available on request from the corresponding author.
